# Chikungunya virus superinfection exclusion is mediated by a block in viral replication and does not rely on non-structural protein 2

**DOI:** 10.1371/journal.pone.0241592

**Published:** 2020-11-12

**Authors:** Jeremy Boussier, Laura Levi, James Weger-Lucarelli, Enzo Z. Poirier, Marco Vignuzzi, Matthew L. Albert

**Affiliations:** 1 Immubiology of Dendritic Cells unit, Institut Pasteur, Paris, France; 2 Inserm U1223, Institut Pasteur, Paris, France; 3 École doctorale Frontières du Vivant, Université Paris Diderot, Paris, France; 4 Viral Population and Pathogenesis Unit, Institut Pasteur, Paris, France; 5 Department of Biomedical Sciences and Pathobiology, Virginia Tech, VA-MD Regional College of Veterinary Medicine, Blacksburg, VA, United States of America; 6 Immunobiology Laboratory, The Francis Crick Institute, London, United Kingdom; 7 Insitro, South San Francisco, CA, United States of America; University of Tennessee Health Science Center, UNITED STATES

## Abstract

Superinfection exclusion (SIE) is a process by which a virally infected cell is protected from subsequent infection by the same or a closely related virus. By preventing cell coinfection, SIE favors preservation of genome integrity of a viral strain and limits its recombination potential with other viral genomes, thereby impacting viral evolution. Although described in virtually all viral families, the precise step(s) impacted by SIE during the viral life cycle have not been systematically explored. Here, we describe for the first time SIE triggered by chikungunya virus (CHIKV), an alphavirus of public health importance. Using single-cell technologies, we demonstrate that CHIKV excludes subsequent infection with: CHIKV; Sindbis virus, a related alphavirus; and influenza A, an unrelated RNA virus. We further demonstrate that SIE does not depend on the action of type I interferon, nor does it rely on host cell transcription. Moreover, exclusion is not mediated by the action of a single CHIKV protein; in particular, we observed no role for non-structural protein 2 (nsP2), making CHIKV unique among characterized alphaviruses. By stepping through the viral life cycle, we show that CHIKV exclusion occurs at the level of replication, but does not directly influence virus binding, nor viral structural protein translation. In sum, we characterized co-infection during CHIKV replication, which likely influences the rate of viral diversification and evolution.

## Introduction

RNA viruses achieve genome diversification through a fast mutation rate and a propensity for recombination between different genomes. This latter phenomenon necessitates the infection of a cell by at least two genomes and is therefore dependent on the potential for cellular co-infection. In this context, superinfection exclusion (SIE, also termed homologous interference), a mechanism by which the infection by a first virus inhibits the infection of a second, typically related, virus, is relevant to genome diversification and virus evolution. SIE has been observed for a number of plant [1–3] and animal viruses [4–7], *in vitro* and *in vivo*, including important human pathogens [8–10]. From an evolutionary standpoint, SIE was proposed to be beneficial for the virus: preventing competition for cellular factors; favoring infection of yet uninfected cells; and limiting replication of defective genomes (i.e., genomes lacking key coding sequences to replicate by themselves). From a practical standpoint, this phenomenon, sometimes termed cross-protection, was used in agricultural practice, by purposefully infecting plants with a mild isolate to protect from a future, more severe, related virus [11]. In the context of SIE, infection by a challenge virus was shown to be impacted at different levels, depending on the virus under study: attachment [9,12], penetration [13], replication [4,14] or viral protein translation [8]. Yet, when one step impacted by SIE has been uncovered, potential blocks at a later step in the viral life cycle have been challenging to investigate.

Chikungunya virus (CHIKV), a member of the genus *Alphavirus*, family *Togaviridae*, is an arbovirus transmitted by *Aedes* mosquitoes, and the causative agent of chikungunya fever, characterized by high fever, rigors, headache, rash and joint pain, which in some persons can result in chronic debilitating disease [15]. Its genome is composed of a single positive-strand RNA molecule of ~12 kb, with two open reading frames, one coding for non-structural proteins (including the RNA-dependent RNA polymerase, also termed replicase), and one coding for the structural proteins (envelope and capsid proteins). Upon binding, CHIKV is internalized, then virus–host cell membrane fusion allows the release of the viral genome in the cytoplasm. Next, the non-structural proteins (nsP1 to 4) are translated, segmented by auto-cleavage and assembled into a replication complex, which catalyzes the synthesis of negative strand full-length genomic RNA that serves as a template for both genomic (49S) and subgenomic (26S) RNAs. Upon 26S RNA translation and cleavage of structural proteins, viral assembly can take place before the virus buds at the cell membrane [16]. This entire cycle requires 6–8 h *in vitro* and in many instances initiates both host innate stress responses, including type I interferon production [17], and triggers cell death pathways [18] that may lead to the demise of infected cells.

While CHIKV SIE had not been investigated experimentally, some is known about related alphaviruses, namely Sindbis virus (SINV) and Semliki Forest virus (SFV). SINV was shown to exclude itself in baby hamster kidney cells [4,5] and in mosquito cells [19,20], and it was hypothesized that the viral protease non-structural protein 2 (nsP2) acts to cleave incoming non-structural polyproteins, thus preventing secondary virions from synthesizing negative-strand RNA [21]. SFV was shown to inhibit future infection by itself, but not other viruses such as influenza A virus (IAV) [13]. SFV exclusion occurred at the level of binding and internalization [13], and was partially dependent on nsP2 [22], as an SFV mutant for nsP2 did not exclude as well as its wild-type counterpart.

Herein, we document and characterize SIE following CHIKV infection. We show that CHIKV inhibits replication of subsequent CHIKV challenge, also excludes SINV and IAV replication, revealing evidence of a cross-family SIE mechanism for alphaviruses. Notably, CHIKV SIE is independent of type I interferon response and host cell transcription. Finally, we show that CHIKV SIE differs mechanistically from SFV SIE, as it does not rely on nsP2, nor does CHIKV impact binding of challenge virus. Instead, CHIKV SIE is mediated by modulation of viral RNA replication of the challenge virus, while leaving structural protein translation unimpacted. These findings may ultimately help define novel strategies for interfering with CHIKV infection.

## Results

### Chikungunya virus superinfection exclusion is broad and not restricted to alphaviruses

**Fig 1:**
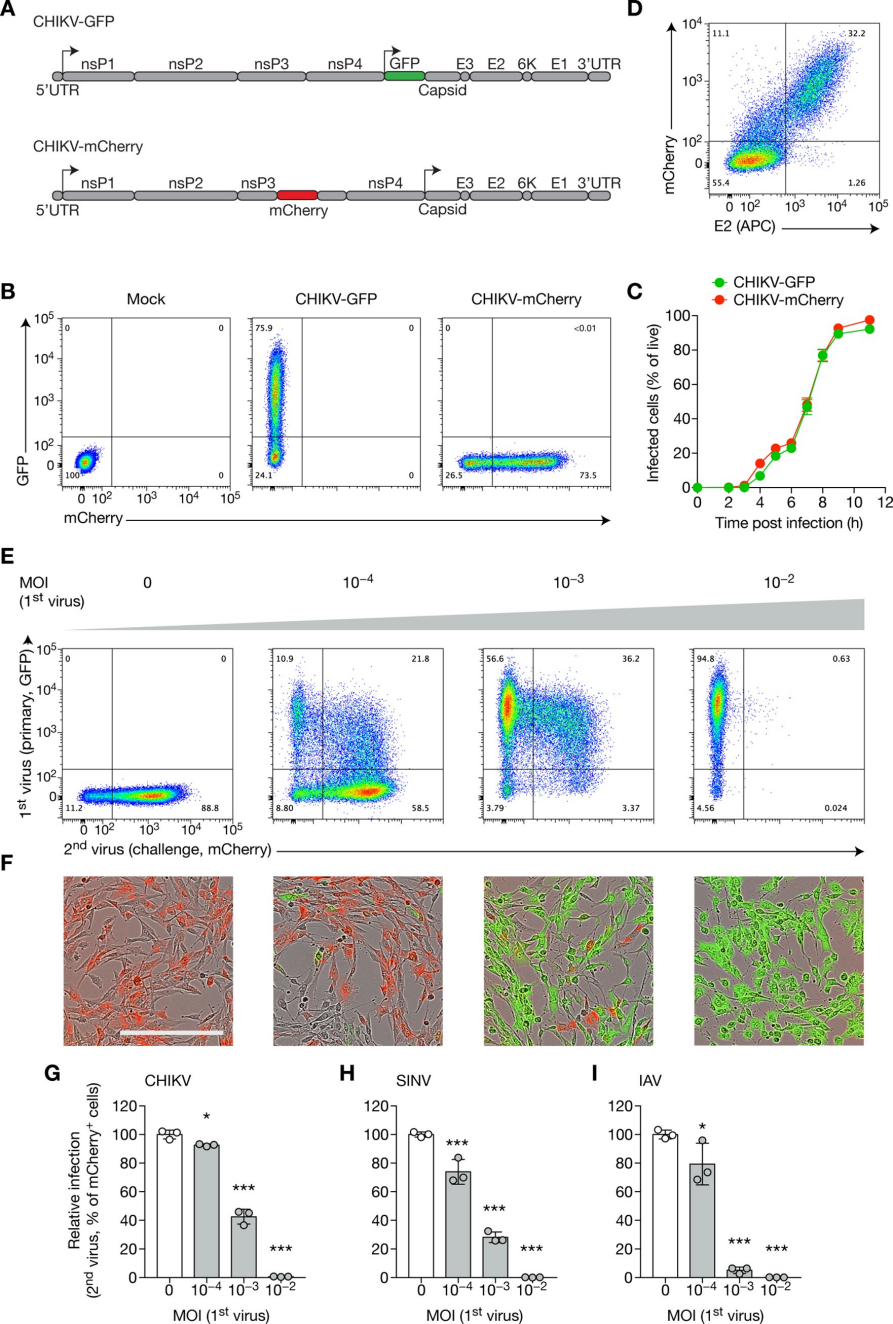
CHIKV superinfection exclusion is not restricted to alphaviruses. (**A**) Schematic of CHIKV genome showing non-structural proteins (nsP1–4), structural proteins (E1–3, capsid, 6K) and 5’ and 3’ UTR (untranslated regions). The 5’ genomic, and downstream subgenomic, promoters are indicated by arrows. Reporter viruses generated from the Indian Ocean lineage include: one with a GFP sequence flanked after the subgenomic promoter (CHIKV-GFP); and a strain encoding a fused nsP3-mCherry protein. (**B**, **C**) BHK cells were infected with CHIKV-GFP or CHIKV-mCherry at an MOI of 1, and GFP and mCherry levels were measured by flow cytometry. Representative flow cytometry plots (B) and percentage of infected cells over time (C) are shown. (**D**) BHK cells were infected with CHIKV-mCherry at an MOI of 10^−4^ for 24 h, then harvested and stained for E2. (**E**–**G**) BHK cells were infected with CHIKV-GFP at the indicated MOI for 16 h, then with CHIKV-mCherry at an MOI of 1 for 8 h, then harvested for flow cytometry analysis (E, quantified in G) or followed by intra incubator microscopy (F). Percentage of infection (G) was normalized by naive control mean. Scale, 300 μm. (**H**, **I**) BHK cells were infected with CHIKV-mCherry for 16 h at the indicated MOI, then infected with SINV-GFP at an MOI of 1 (H) or with IAV at an MOI of 3 (I) for 8 h. Cells were harvested and analyzed directly by flow cytometry (H) or were intracellularly stained with anti-NP-FITC (I) antibody before analysis. Bars indicate mean ± SD of biological triplicates, and data are representative of at least two independent experiments. NS, not significant; *p < 0.05, ***p < 0.001 (one-way analysis of variance followed by Dunnett’s post-test).

To study SIE triggered by CHIKV, we used two reporter viruses derived from an Indian Ocean CHIKV strain: one coding for GFP, inserted immediately after the subgenomic promoter, thus making GFP expression a reporter of subgenomic transcription [23]; and one coding for the mCherry protein, inserted to generate a fusion protein with nsP3, making it a genomic reporter [24] (Fig 1A). Both displayed similar kinetics of infection of baby hamster kidney (BHK) cells, as monitored by flow cytometry (Fig 1B, 1C and S1 Fig). To assess the sensitivity of fluorescence detection compared to conventional antibody-staining, we infected BHK cells at a multiplicity of infection (MOI) of 10^−4^ for 24 h and stained them with anti-E2 antibody followed by flow cytometry (Fig 1D). Fluorescent marker was more sensitive than E2 staining in determining cells with active replication, with few E2^+^ mCherry^−^(1.26%) compared to double positive cells (32%) and E2^–^ mCherry^+^ cells (11.1%). This result also suggests the scarcity of mutations in the fluorescent gene, and the low rate of false negatives induced by flow cytometry, even throughout the course of a 24-h infection.

While most studies focus on SIE triggered post-infection with high or very high MOI (up to 10–100), we decided to study lower, more physiologically relevant MOIs. Additionally, instead of focusing on very short times between infections (15–30 min), we infected cells for longer times before challenging them with a second virus, to better mimic real-life sequential infections, which rarely occur throughout a 1 h period, and to take into account the role of potential factors participating in SIE when the infections occur several hours apart. Therefore, to assess the capacity of CHIKV to exclude itself, we infected BHK cells with CHIKV-GFP at low multiplicity of infection (MOI) for 16 h (approximately two viral cycles) then CHIKV-mCherry at high MOI for 8 h (one viral cycle) and followed protein upregulation by flow cytometry and real-time microscopy. Herein, we will refer to the second virus used as the “challenge” virus, an experimental means of assessing the ability of the initial virus to establish a state of SIE. Confounding effects of dead cells were addressed by the flow cytometry gating strategy (S1 Fig). Naive cells were highly susceptible to CHIKV-mCherry infection, whereas infected cells were protected from the challenge virus, as a function of the MOI of the initial input virus (Fig 1E–1G). Replication of the challenge virus was almost fully inhibited in cells having been initially infected using an MOI of 0.01. Notably, a non-trivial population of double positive cells infected by both GFP and mCherry viruses was observed at low MOIs, showing that superinfection was possible with low input of first virus(es). However, this population completely disappeared as the MOI of the first virus increased. Interestingly, at the highest MOI, all cells showed complete exclusion, although they displayed a diverse range of GFP expression. To establish whether active replication in the host cell is a determinant of SIE, we next inactivated CHIKV by UV irradiation. CHIKV inactivation completely abolished exclusion, suggesting a strict requirement for active viral replication of the initial virus (S2A Fig), as described in other virus models [5,12,25]. To exclude the impact of the reporter system, we confirmed that reversing the order of the two viruses gave similar results (S2B–S2D Fig).

As SIE is often described as a mechanism affecting “homologous” viruses (typically viruses belonging to the same family), we tested whether CHIKV could exclude infections by Sindbis virus (SINV) and influenza A virus (IAV). As expected, CHIKV-infected cells were protected from infection by the alphavirus SINV (Fig 1H). Surprisingly, IAV, a segmented negative-strand virus belonging to the *Orthomyxoviridae* family, was also excluded by CHIKV (Fig 1I). Together, these data indicate that SIE triggered by CHIKV is a cross-family phenomenon.

### CHIKV SIE is independent of the action of type I interferon

**Fig 2:**
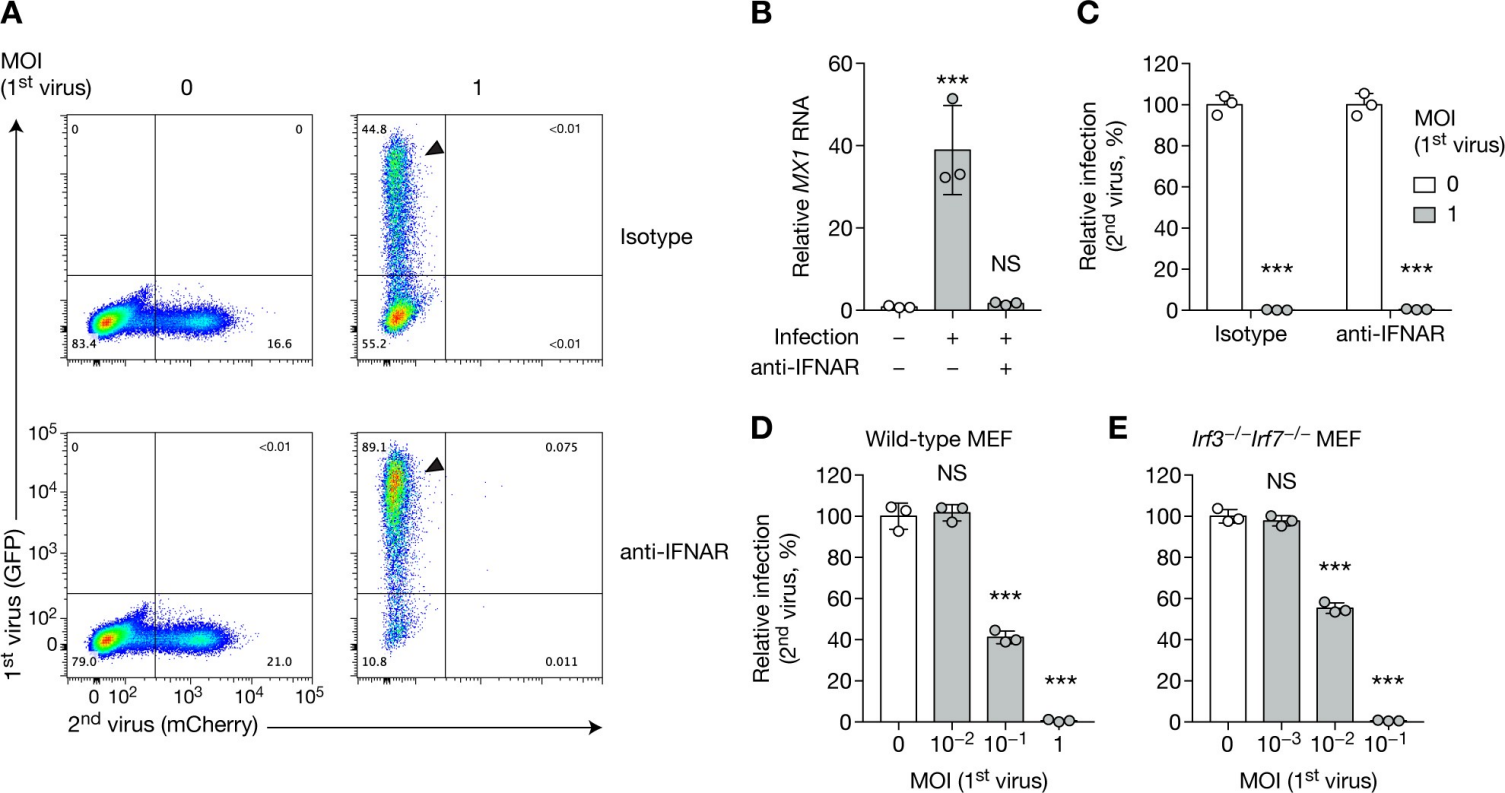
CHIKV SIE is independent on type I interferon. (**A**–**C**) HFFs were treated with human IFNAR blocking antibody or isotype control at 5 μg/mL for 1 h, then were infected with CHIKV-GFP for 24 h at the indicated MOI, followed by CHIKV-mCherry at MOI 1 for 24 h. Blocking antibody treatment was maintained throughout the experiment. Representative flow cytometry plots (A) and quantification of infected cells (C) and are shown. Arrows highlight the difference in GFP infection between isotype control and blocking antibody treated samples. *MX1* RNA levels were assessed by RT–qPCR (B). (**D**, **E**) WT (D) or *Irf3*^−/−^*Irf7*^−/−^MEF cells (E) were infected with CHIKV-GFP at the indicated MOI for 16 h, then with CHIKV-mCherry at MOI 5 (WT) or 3 (*Irf3*^−/−^*Irf7*^−/−^) for 8 h, and subsequently analyzed by flow cytometry. Bars indicate mean and SD of biological triplicates, and data are representative of at least two independent experiments. NS, not significant; ***p < 0.001 (one-way analysis of variance followed by Dunnett’s post-test).

One obvious explanation might relate to the general antiviral host mechanisms that are triggered by infection. Namely, type I interferon (IFN) is triggered by CHIKV infection [17,26,27], which could account for the protection of cells from subsequent viral challenge [17,28]. While we demonstrate the existence of CHIKV-induced SIE in BHK cells, which are IFN incompetent, it was important to formally test the role of IFN or IFN-stimulated genes (ISGs) in SIE. IFN-competent primary human foreskin fibroblasts (HFF cells) were infected in the presence or absence of interferon-α/β receptor blocking antibody (anti-IFNAR). Cells infected in the presence of anti-IFNAR were more sensitive to infection, with 89% of cells infected by CHIKV as compared to 45% of IFNAR-responsive cells (Fig 2A, arrows). Increased *MX1* RNA levels served as an additional control for the efficiency of IFNAR blockade (Fig 2B). Preventing ISG expression did not prevent SIE, as the challenge virus did not show expression of mCherry in either condition (Fig 2A and 2C). Additionally, type I and type III IFN production was blunted in mouse embryonic fibroblasts (MEF cells) via the deletion of the IRF3 and IRF7 transcription factors. *Irf3*^−/−^*Irf7*^−/−^ double knockout MEF cells displayed CHIKV SIE similar to what was observed in wild-type MEF cells (Fig 2D and 2E). These data validate the findings in BHK cells, and indicate that CHIKV SIE mechanism of action is IFN independent.

### CHIKV SIE is a cell-intrinsic mechanism independent of host cell transcription

**Fig 3:**
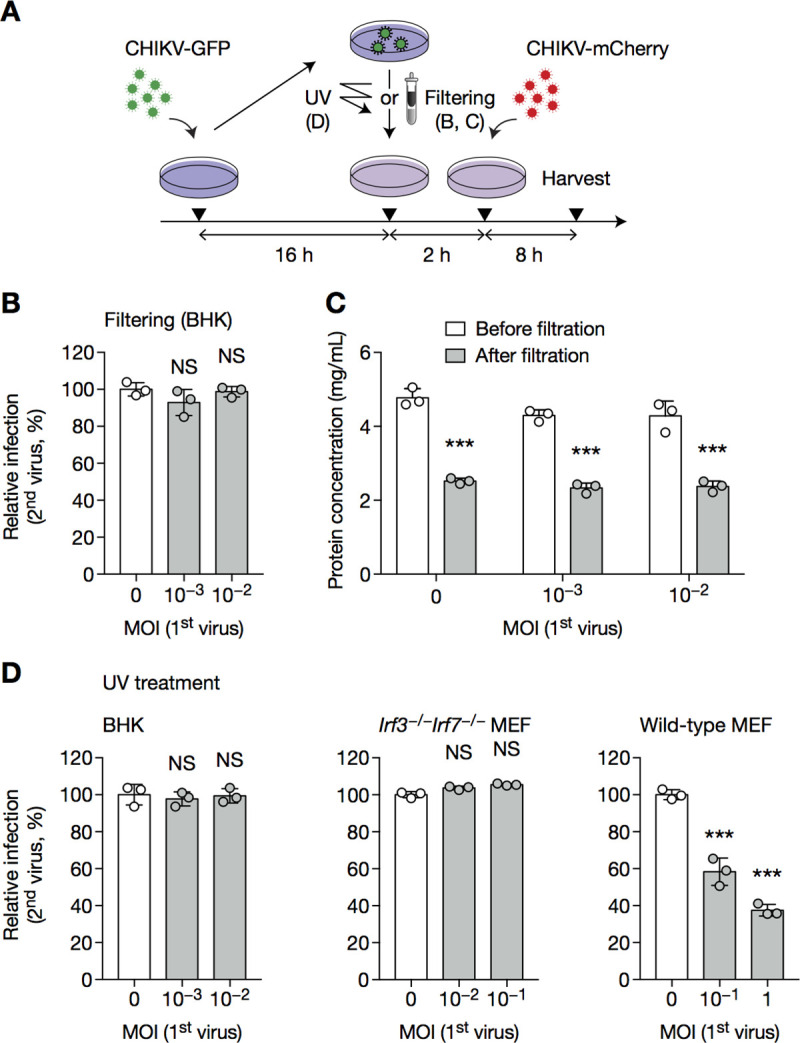
CHIKV SIE is a cell-intrinsic mechanism. BHK cells, WT or *Irf3*^−/−^*Irf7*^−/−^MEF cells were infected with CHIKV-GFP at the indicated MOI (**A**), then supernatant was subject to filtering (**B**, **C**) or UV irradiation (**D**) and overlaid onto fresh cells, which were challenged 2 h later with CHIKV-mCherry at an MOI of 1 (BHK), 5 (wild-type MEF) or 3 (*Irf3*^−/−^*Irf7*^−/−^ MEF) for 8 h, then harvested and analyzed by flow cytometry. Protein concentration before and after filtration was assessed in C. Bars indicate mean ± SD of biological triplicates, and data are representative of two independent experiments. NS, not significant; ***p < 0.001 (one-way analysis of variance followed by Dunnett’s post-test).

In the above infection/challenge experiments, it is notable that GFP^−^ cells were protected from the challenge mCherry virus (Fig 1E, MOI 0.01). Although IFN does not participate in CHIKV SIE (Fig 2), an alternative soluble factor might be protecting uninfected cells. To test this hypothesis, we infected BHK cells with increasing MOIs of CHIKV-GFP for 16 h, then collected the culture supernatant. We subsequently subjected the supernatant to ultrafiltration, sufficient to remove free virus; and overlaid the resultant virus-free supernatant onto fresh cells. After 2 h, the cells were challenged with CHIKV-mCherry (Fig 3A). Cells overlaid with supernatant from infected cells were as susceptible to infection as those overlaid with supernatant from naive cells, suggesting that a soluble factor did not account for the exclusion (Fig 3B). One caveat of this experiment was that filtering reduced protein concentration by ~50%, most likely due to the loss of protein aggregates that could not pass through the filter (Fig 3C). We therefore used an alternative method to test the same hypothesis: instead of filtering the input virus, we briefly UV-irradiated the culture supernatant prior to overlaying it onto fresh cells, and subsequent challenge (Fig 3A). BHK cells and *Irf3*^−/−^*Irf7*^−/−^ MEF cells (both IFN-incompetent) were sensitive to the challenge infection, indicating that soluble factors do not account for SIE (Fig 3D). By contrast, cells overlaid with supernatant from infected WT MEF cells were still partially protected due to the production of IFN-β, suggesting that UV did not damage effector proteins (Fig 3D, compare with Fig 1G).

**Fig 4:**
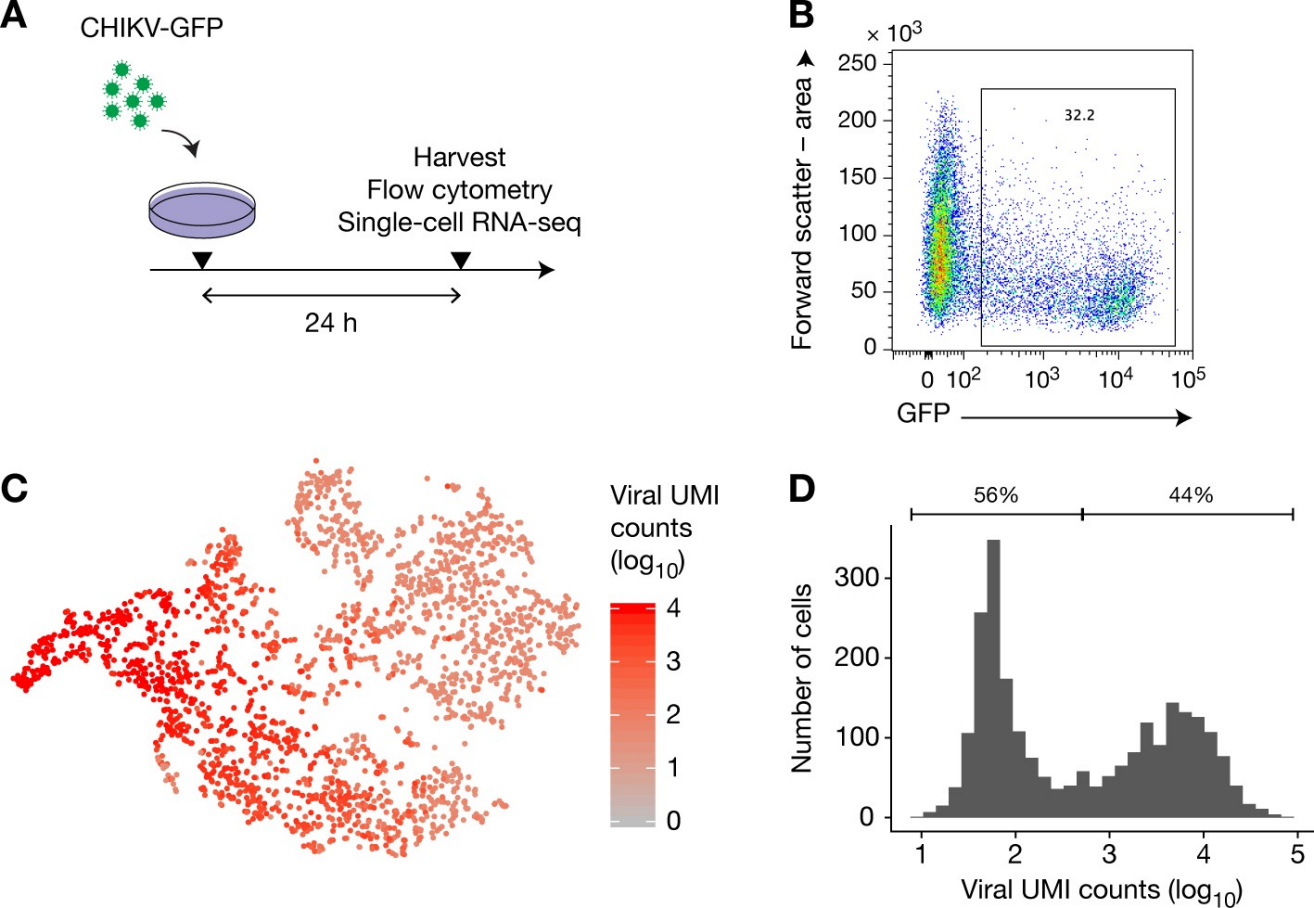
After primary infection, all cells contain viral RNA. (**A**) HFFs were infected with CHIKV-GFP at MOI 1 for 24 h, then harvested and separated into two samples for analysis by flow cytometry and single-cell RNA-sequencing using 10x genomics Single Cell 3’. (**B**) Flow cytometry analysis of the infected cells. (**C**) T-SNE analysis of the RNA-sequencing data. (**D**) Distribution of the number of CHIKV unique molecular identifiers (UMI) per cell.

The cell-intrinsic nature of SIE suggests that only cells infected with the first virus can be protected from a future infection. Paradoxically, we observed that during SIE experiments, GFP^−^ cells (i.e., cells uninfected by the first virus) are protected from challenge infection (see Fig 1D, MOI 0.01 for example). To resolve these apparently contradictory findings, we next examined whether these cells were indeed actually uninfected, or if they contained viral proteins at too low copy numbers to be detectable by flow cytometry. To this end, we infected HFF cells with CHIKV-GFP at an MOI of 1 for 24 h (Fig 4A), and analyzed the cells by flow cytometry and single-cell RNA sequencing. We found that, although only 32% of cells were found GFP^+^ by flow cytometry (Fig 4B), all cells contained at least 8 molecules of RNA (with different unique molecular identifiers) that aligned to the CHIKV genome (Fig 4C and 4D). Since it was estimated that the 10x reagent chemistry captured approximately 6.7–8.1% of mRNA transcripts [29], cells with ≥8 sequenced UMIs likely carry several hundreds of viral RNA molecules. These data suggest that all cells contained virus, and that exclusion of GFP^−^cells cannot exclude a cell-intrinsic mechanism. While cells were exposed to trypsin digestion and subsequent extensive washing prior to 10x processing, we cannot not formally exclude that the detected RNA may have been derived from extracellular viruses bound to the cell. Replication of this finding and additional single molecules studies will be required to further assess the presence of viral RNA in GFP^−^cells.

**Fig 5:**
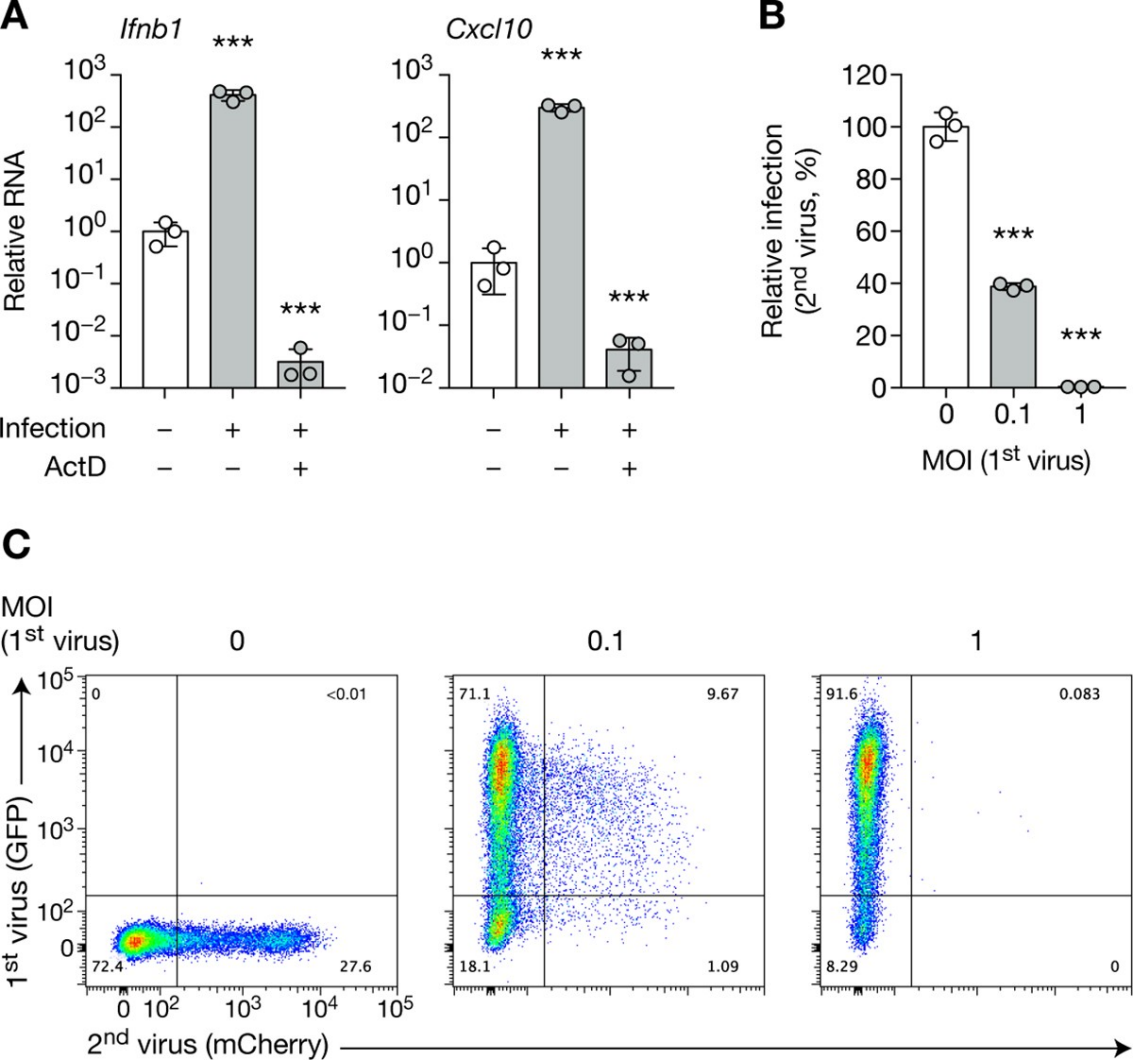
*De novo* host cell transcription is dispensable for the establishment of CHIKV SIE. BHK cells, pre-treated with ActD or DMSO, were infected with CHIKV-GFP for 8 h at the indicated MOI, then with CHIKV-mCherry at MOI 1 for 8 h. Quantification of *Ifnb1* and *Cxcl10* RNA (**A**), infected cells (**B**) and representative flow cytometry plot (**C**) are shown. Bars indicate mean ± SD of biological triplicates, and data are representative of two independent experiments. NS, not significant; ***p < 0.001 (one-way analysis of variance followed by Dunnett’s post-test).

We thus proposed that CHIKV SIE relies on a cell-intrinsic mechanism, which could arise either via factors present before infection, or those newly transcribed after the infection of the primary virus. To determine which of these two mechanisms contribute to SIE, we tested the requirement of *de novo* transcription. We infected BHK cells in the presence or absence of actinomycin D (ActD), a specific inhibitor of the host DNA-dependent RNA polymerase, which does not directly inhibit viral polymerases. ActD treatment completely blocked de novo cellular transcription, as cells infected with CHIKV were unable to upregulate *Ifnb1* and *Cxcl10* RNA (Fig 5A). Cells treated with ActD, although less infectable than untreated cells (compare Figs 1E and 5C), could still support MOI-dependent SIE (Fig 5B and 5C), indicating that *de novo* cellular transcription is not necessary to induce SIE.

### Single viral protein expression does not confer protection observed in SIE

To examine the role for viral-mediated exclusion, we applied a reductionist approach and tested the impact of single viral protein expression. Support for this hypothesis comes from prior work on Semliki Forest virus (SFV), a close relative of CHIKV, which was shown to exclude a challenge SFV infection in a mechanism partially dependent on non-structural protein 2 (nsP2). Notably, Ehrengruber *et al*. generated an SFV mutant (SFV(PD)) with two amino-acid changes in its nsP2 sequence, which was less cytopathic and allowed much higher transgene expression than wild-type SFV (WT SFV) [22]. Interestingly, infection with the SFV(PD) followed by superinfection with WT SFV resulted in a weaker SIE phenotype, as indicated by a greater proportion of co-infected cells compared to when WT SFV was used as the primary virus.

To examine the applicability of this finding to CHIKV SIE, we compared CHIKV and SFV nsP2 sequences, and found 84% similarity at the amino-acid sequence with identity at the two positions identified in SFV to be critical for SIE. We incorporated the homologous mutations S259P and R650D in the CHIKV-GFP genome (termed CHIKV-GFP(PD), S3A Fig). By contrast with the findings in SFV, CHIKV-GFP(PD) displayed wild-type growth kinetics in BHK cells (S3B Fig). Moreover, when tested, CHIKV-GFP(PD)-infected cells showed robust exclusion of challenge virus (S3C Fig), with no change in the number of double-positive cells (S3D Fig). These experiments demonstrated that, contrary to SFV SIE, CHIKV does not rely on the aforementioned nsP2 residues.

**Fig 6:**
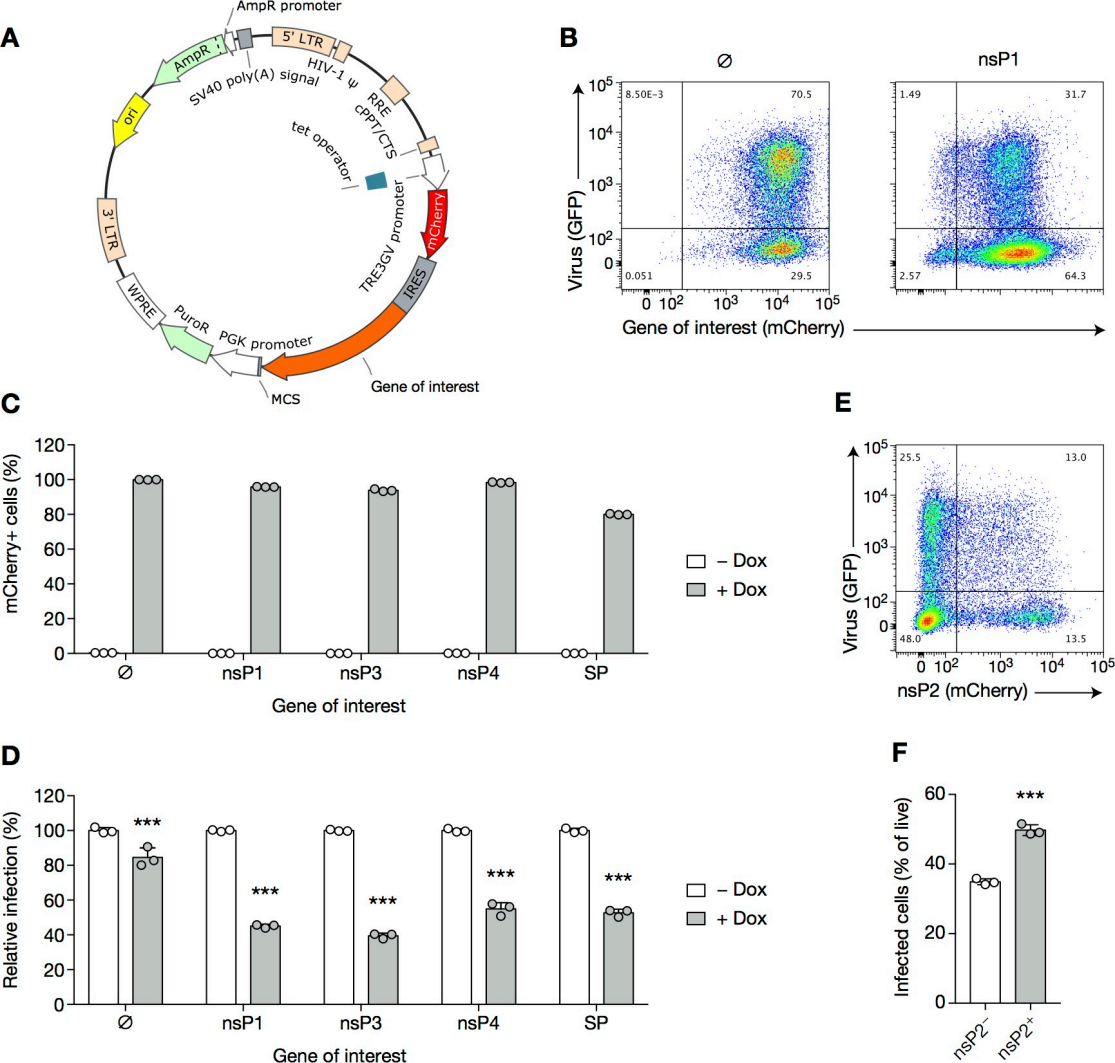
CHIKV SIE is not mediated by a single viral protein. (**A**) Schematic representation of the tetracycline-inducible construct. PuroR, puromycin resistance. LTR, long terminal repeat; HIV-1 ψ, packaging signal of human immunodeficiency virus type 1; PGK, mouse phosphoglycerate kinase 1; PuroR, puromycin resistance (puromycin N-acetyltransferase); AmpR, ampicillin resistance (β-lactamase); cPPT/CTS, central polypurine tract and central termination sequence of HIV-1; WPRE, woodchuck hepatitis virus post-transcriptional regulatory element; RRE, Rev response element of HIV-1; MCS, multiple cloning site, where genes of interest were cloned; IRES, internal ribosome entry site; ori, origin of replication. (**B**–**D**) 3T3 cells stably co-expressing the transactivator protein (3T3 transactivator) and the tetracycline-inducible construct described in (A) were treated with doxycycline (1 μg/mL) or left untreated for 16 h, then infected with CHIKV-GFP for 8 h at an MOI of 4, and subsequently harvested and analyzed by flow cytometry. Representative flow cytometry plots (B), percentages of cells expressing the transgene (C) and percentage of infected cells (D) and are displayed. SP, structural protein. (**E**, **F**) 3T3 cells stably expressing the transactivator protein (3T3 transactivator) were transfected with the construct containing nsP2 gene. Twenty-four hours post-transfection, cells were plated and treated or not with 1 μg/mL of doxycycline for 16 h, then challenged with CHIKV-GFP at an MOI of 4. Eight hours post-infection, they were harvested and analyzed by flow cytometry. Representative flow cytometry plot (E) and percentage of infected cells in the mCherry^+^and mCherry^−^populations (F) are shown. Bars indicate mean ± SD of biological triplicates and data are representative of two independent experiments. ***p < 0.001 (two-way ANOVA followed by Sidak’s post-test (C) or unpaired *t*-test (D)).

To assess the potential contribution of other viral proteins in establishing SIE, we generated stable 3T3 cell lines expressing inducible nsP1, nsP3, nsP4 or all structural proteins (Fig 6A). Cells were treated with doxycycline and challenged with CHIKV-GFP, using untreated cells as a negative control. Expression of the protein(s) of interest was robust, with nearly 100% of cells showing induced expression (as per mCherry expression, Fig 6B and 6C), to the level observed in SIE experiments (compare with S2C Fig), yet only 40–60% of the cells were protected from CHIKV-GFP infection (Fig 6D). Hence, none of the cell lines showed protection to the extent (~100%) achieved by live primary CHIKV infection (Fig 1D). Why partial protection is achieved to about the same extent by any of the CHIKV proteins is unclear, and we hypothesize a role for extreme overexpression of these proteins, in a non-physiological way.

We did not succeed in obtaining cell lines expressing inducible nsP2, likely because of its toxicity to cells due to its known inhibition of cellular transcription [30]. We considered this a key question as the mutant virus (reported in S3 Fig) does not exclude a role for other nsP2 protein domains. To circumvent the technical issue regarding cell line generation, we transiently transfected nsP2 into 3T3 cells, and infected them in the presence of doxycycline. This achieved expression of nsP2 in >20% of cells, as assessed by mCherry expression (Fig 6E). However, mCherry^+^ cells were even more susceptible to infection as compared to mCherry^−^ cells, suggesting that nsP2 alone does not account for CHIKV SIE (Fig 6F). As a single viral protein could not be assigned to the establishment of an SIE state, we turned to the more integrated processes enacted by a virion during infection and replication.

### Primary virus does not impact binding

**Fig 7:**
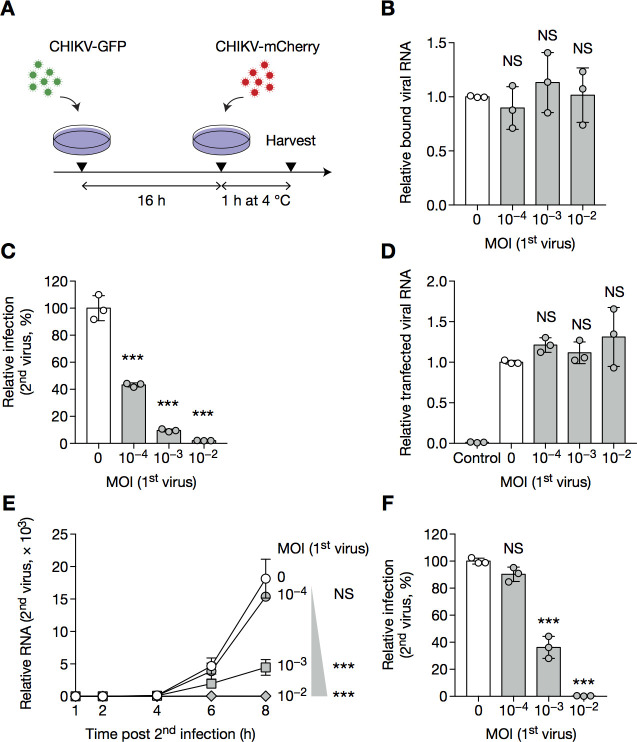
Primary virus inhibits replication of the challenge virus. (**A**–**B**) BHK cells were infected with CHIKV-GFP for 16 h at the indicated MOI, then with CHIKV-mCherry at MOI 1 for 1 h at 4°C and harvested for RT–qPCR specific for mCherry. (**C**, **D**) BHK cells were infected with CHIKV-GFP for 16 h at the indicated MOI, then transfected with *in vitro* transcribed RNA coding for CHIKV-mCherry. 12 h post-transfection, cells were harvested and analyzed by flow cytometry (C); 4 h post-transfection, transfection efficiency was controlled by RT–qPCR (D). Control wells were overlaid with transfection mix for 5 min at room temperature, to assess background noise after washing. (**E**, **F**) BHK cells were infected with CHIKV-GFP at the indication MOI for 16 h then CHIKV-mCherry at MOI 1 for the indicated time, and harvested for RT–qPCR specific for mCherry (E) or analyzed by flow cytometry (F) to provide the protein data for this particular day of experiment. Bars indicate mean ± SD of biological triplicates, and data are representative of at least two independent experiments. NS, not significant; *p < 0.05, **p < 0.01, ***p < 0.001 (one-way analysis of variance followed by Dunnett’s post-test).

Disruption of challenge infection may occur at various stages of the viral life cycle. While it was shown that SFV exclusion partly occurred at the level of attachment [13], and may also involve inhibition of challenge virion penetration, nothing is known for CHIKV SIE. To track entry and replication of the challenge virus, we designed PCR primers specific for the GFP and mCherry reporters, providing a means to distinguish primary and challenge virus genomes prior to initiating protein translation (S4 Fig). To determine whether SIE acts at the level of virus binding, cells were infected or not with CHIKV-GFP and challenged with CHIKV-mCherry for 1 h at 4°C, allowing for engagement of the plasma membrane entry receptor(s), but preventing internalization (Fig 7A). Cells were then washed extensively and lysed, followed by RNA extraction and RT–qPCR using primer–probe sets specific for mCherry. Naive and pre-infected cells bounded CHIKV-mCherry at similar levels, indicating that exclusion does not occur at the level of viral attachment (Fig 7B).

### CHIKV SIE blocks the replication of the challenge virus

Next, we tested whether CHIKV SIE blocks the challenge virus at a later step of its life cycle. To do so, we bypassed viral entry steps (attachment, internalization and penetration) of challenge infection by transfecting cells with *in vitro* transcribed viral RNA coding for CHIKV-mCherry. Such transfection did not overcome exclusion (Fig 7C), indicating that SIE impairs distal steps in the life cycle of the challenge virus. The differences observed were not due to transfection efficiency, as assessed by the RNA levels 4 h post-transfection (Fig 7D). Again, reversing the order of the two viruses gave similar results (S5A and S5B Fig).

We additionally monitored replication of the challenge virus in naive and infected cells. We found that RNA replication was impacted in an MOI-dependent manner (Fig 7E), reflecting the expression of protein data obtained by flow cytometry (Fig 7F), and indicating that CHIKV SIE acts by inhibiting challenge virus replication.

### Viral structural protein translation is not impacted in excluded cells

Cells infected at an MOI of 0.01 supported modest but measurable replication of the challenge virus, as observed when data are plotted on a logarithmic scale (S5C Fig); note a ~60-fold increase in RNA expression (S5D Fig). By contrast, flow cytometry analysis revealed virtually no mCherry-positive cells (marker for the challenge virus) (Fig 1E). This raised the question of whether there was a downstream block at the level of protein translation.

**Fig 8:**
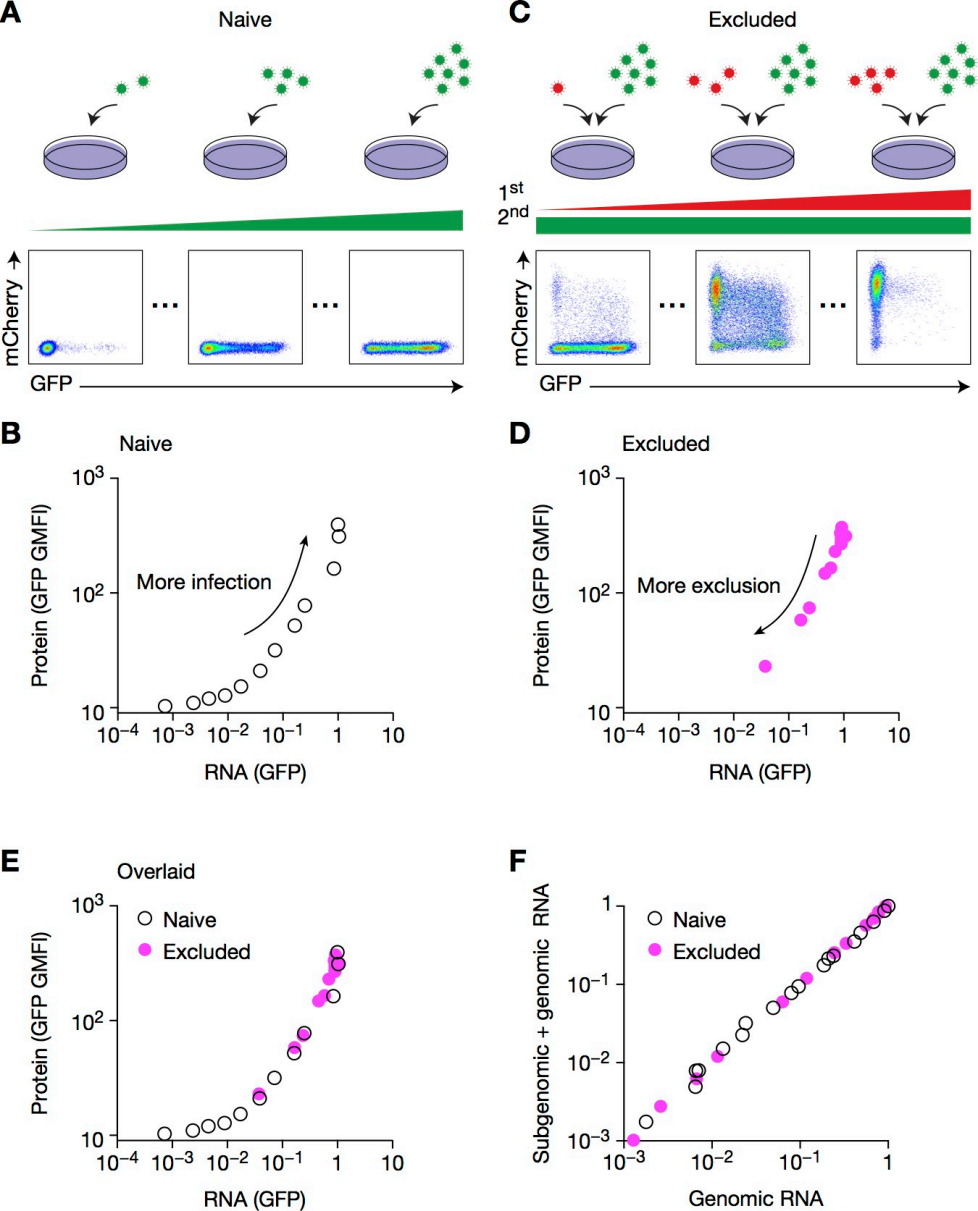
Viral structural protein translation is not impacted in excluded cells. (**A**, **B**) BHK cells were infected with CHIKV-GFP at increasing MOIs for 8 h, then harvested for both flow cytometry and RT–qPCR. Representative flow cytometry plots (A), and quantification of mCherry geometric mean fluorescence intensity (GMFI) and mCherry RNA (B) are shown. (**C**, **D**) BHK cells pre-infected with CHIKV-mCherry at increasing MOIs for 16 h then CHIKV-GFP at MOI 1 for 8 h were harvested and depicted as in A and B. (**E**) Overlaying of B and D. (**F**) BHKs were infected as in E, and were harvested and analyzed by RT–qPCR with primer–probe sets specific for GFP or for the genomic form of CHIKV-GFP (referred to as GFP-genomic), whose respective sensitivity and specificity were tested in S3 Fig. Data are representative of two independent experiments.

To test this hypothesis, we designed an experiment to assess the relationship between viral RNA and protein expression during a first or a second infection. To this end, naive cells were infected with increasing MOIs of CHIKV-GFP, and RNA and protein levels were assessed by RT–qPCR and flow cytometry, respectively (Fig 8A and 8B). As a second data set, cells were first infected with increasing MOIs of CHIKV-mCherry and challenged with CHIKV-GFP at a fixed high MOI, thereby defining the RNA-to-protein expression relationship in excluded cells (Fig 8C and 8D). Notably, the graphs for singly infected (naive) and doubly infected (excluded) cells overlaid perfectly (Fig 8E), indicating that the relationship between RNA expression and protein translation is similar in primary and challenge infections. In other words, the primary infection does not impact protein translation. As the GFP reporter is subgenomic, this led us to conclude that translation of full-length genomic RNA is not impacted. Of note, the ratios of genomic and subgenomic RNAs were similar in primary and challenge virus infections (Fig 8F). This approach constitutes a novel method to test the impact of SIE on further steps of the viral life cycle. Together, these results indicate that SIE acts solely on challenge viral replication, and not by the inhibition of structural protein translation.

## Discussion

In this paper, we systematically characterized SIE triggered by CHIKV infection. It is, to our knowledge, the first report of CHIKV SIE, and our findings indicate that CHIKV is unique as compared with what was previously reported for the alphavirus SFV. Although SFV SIE was shown to occur at the level of viral attachment [13], binding of the challenge virus is not impacted by CHIKV infection, which instead blocks replication of the second virus. Furthermore, SFV SIE did not prevent future IAV exclusion, as CHIKV did. Last, nsP2 was shown to be partially mediating SFV SIE, as an SFV mutant for nsP2 induced the same global level of exclusion as wild-type SFV, but with much fewer double-positive cells [22]. This suggested a cell-intrinsic phenomenon whereby cells infected by the first virus are protected, and this protection is compensated for by a higher infection rate of naive cells (uninfected by the first virus), resulting in the same overall rate of infection by the second virus. The most straightforward explanation is that binding of the challenge virus is impacted only in infected cells, and therefore, for a given amount of viral inoculum, binding is increased in uninfected cells. This hypothesis is consistent with the fact that SFV infection impairs challenge SFV binding. One possibility is that nsP2-mediated transcription inhibition (requiring the nuclear localization signal) prevents the fast renewal of SFV receptors at the surface of the cells, thereby impacting future binding. As CHIKV SIE does not impair binding of the challenge virus, it seems coherent that nsP2 is not involved in CHIKV SIE.

Unlike most SIE studies, our experimental design includes longer time intervals between first and challenge infections. This is motivated by the fact that real-life superinfection rarely occurs within 15–60 min of the first infection, and that such short time intervals may lead to missing out key mechanisms responsible for SIE, in particular those involving the cell response to infection. The offset is that we had to use low MOIs for the first infection, which have the advantage to better mimic real-life infection, but can rapidly lead to asynchronous infections. These differences in experimental design may explain some of the discrepancy between our results and what is known for other alphaviruses, but can also shed light on mechanisms overlooked by previous “very early” SIE studies.

In our review of the literature, we suggest that most studies focused on one proximal discovery (e.g., block in replication), without characterizing later steps whereby viral agents might prevent secondary infection (e.g., translation). We provide a new approach and data-driven strategy to compare protein translation of the challenge virus in naive and infected cells, despite an earlier block at the level of replication. To circumvent the issue of differential RNA loads, we chose to determine the RNA–protein relationship throughout a wide range of replication levels, thus permitting us to disentangle the efficiency of protein translation for a given RNA level and supporting the comparison between naive and excluded cells. To this end, naive cells were infected with increasing MOIs of challenge virus, while excluded cells were subjected to different levels of exclusion. Thus, if exclusion impacted translation, the two curves would split as challenge RNA virus decreases (i.e. as infected cells are more excluded), while perfectly overlapping curves indicate no impact on translation. This method will be well suited to more precisely characterize all steps impacted during challenge infection in future work on SIE.

Mechanistically speaking, we have shown that no single CHIKV protein could fully explain SIE, and although a synergistic effect could not be formally excluded, these data suggest that a cellular response may be at play. We demonstrated that neither type I IFN, nor any soluble factor, could account for SIE, and that *de novo* cellular transcription was dispensable for the induction of SIE. Therefore, other cellular pathways, relying on the direct activation of proteins with basal level of expression, could be at play. In this regard, a good candidate is PKR: it is expressed at basal levels, and upon direct activation by double-strand RNA, triggers the phosphorylation of EIF2α and the subsequent inhibition of host translation [31,32]. While CHIKV subgenomic RNA can be translated independently of EIF2α [33,34], it is possible that genomic RNA from incoming challenge virus requires the EIF2α translation machinery, and is therefore blocked at the first step of its RNA replication cycle. Future work will aim to decipher the different requirements in terms of cellular translational machinery during early infection of these different viruses, and the potential role of PKR in establishing SIE. Additionally, it remains to explain why infection by IAV is also excluded by a primary CHIKV infection, since our data does not exclude the possibility of multiple mechanisms. Because IAV, SINV and CHIKV all possess a poly(A) tail, SIE may rely on an RNA degradation mechanism that preferentially targets RNA carrying poly(A) tail. In this regard, the nonsense mediated decay (NMD) machinery, allowing the detection and degradation of RNA molecules with premature stop codons, may be a good candidate, as it was proposed to be triggered by the too-long distance between stop codons and poly(A) tail [35]. NMD has already been implicated in the recognition of alphaviruses [36], but future work should address whether it can target IAV, and if NMD mediates SIE.

Research on SIE has led to the description of multiple phenomena, a reflection of unique host–virus interdependencies. However, SIE induced by different viruses share common characteristics. First, SIE is for the most part cell-intrinsic, meaning that only cells infected by the first virus are protected from a challenge infection. In our model, cells infected at the highest MOI and that remained GFP^−^(uninfected by the first virus) were still protected from the second infection. We argue that this is consistent with a cell-intrinsic mechanism as GFP^−^ cells contain viral RNA (Fig 4), as assessed by single-cell RNA sequencing. Whether this viral RNA is undergoing partial replication remains to be determined, and it would support a putative therapeutic use of defective viruses as a mechanism for protecting cells from subsequent infection. Alternatively, cells may have a distinct propensity to infection, and accordingly, some cells are resistant to replicative infection, and in turn intrinsically protected from the challenge virus.

A second shared characteristic of SIE is that it occurs post-entry. Although early studies focused on receptor-mediated interference [13,37–39]—whereby a first infection induces downregulation of entry receptors—recent evidence has suggested that even in these viral models, a downstream block exists, and that receptor-mediated interference was only minorly contributing to SIE. Indeed, mutants that were poor downregulators of entry receptors were still capable of inducing exclusion [9,12]. Additionally, direct transfection of challenge virus genetic material did not overcome SIE [14]. In some viral models, the fitness advantage provided by receptor downregulation was in fact proposed to be independent of SIE: for example, HIV downregulation of CD4 in infected cells (initially presumed to be a key determinant of SIE) prevents the formation of non-functional gp120–CD4 complexes at the surface of the released virions, thereby increasing infectivity [40–43].

SIE has been proposed to have evolutionary benefits for the viral population. An optimal rate of co-infection vs. exclusion would enable a balance between genetic diversification (required to adapt to varying selective pressures) and genomic integrity (required to avoid lethal mutagenesis). Notably, SIE limits recombination as well as the potential replication of defective viral genomes (which necessitates helper virus machinery). As both processes participate in the genetic diversification of the virus, SIE may help maintain genetic stability. Zhang *et al*. have proposed another fitness advantage of SIE, which would not be limited to sequential infections. In their model, infection by a virus, after a few hours, not only excludes superinfecting viruses, but also inhibits replication of daughter genomes. Thus, SIE—in this case better termed “progeny exclusion”—ensures that only the parental genome, and not the daughter genomes, is replicated [44]. This would establish a first round of selection as daughter genomes would need to be faithfully packaged and competent for achieving infection in their own right, before being allocated cellular resources for their replication. Accordingly, SIE i) would be a cell-intrinsic mechanism—as the target of the exclusion is the direct progeny of the virus—; ii) would occur at a post-entry step; and iii) would act within one or two viral cycles. While the first two characteristics are commonalities among most viral SIE models, the timing at which exclusion is achieved has not been carefully studied. Interestingly, our findings are consistent with this hypothesis: CHIKV SIE is most probably triggered early post-infection (without the need for *de novo* transcription); it constitutes a cell-intrinsic mechanism, and blocks replication of the challenge virus, at a post-entry step.

If this model holds true, SIE would serve as a means to balance genome diversification and integrity, and avoid the too-fast accumulation of deleterious mutations. Through its impact on recombination, defective genome replication, and mutational rate, SIE may well be at the center of quasispecies swarm regulation. Future work establishing the role of the cellular response in SIE may provide novel insights into the crosstalk between quasispecies dynamics and the cellular response to infection.

## Materials and methods

### Cells

Wild-type and *Irf3*^−/−^*Irf7*^−/−^ MEF cells were obtained from the Lenschow laboratory (Washington University, USA); BHK21, HFF and Vero cells were purchased at ATCC. All cell lines were tested for mycoplasma, and were cultured in complete Dulbecco modified Eagle's minimal essential medium with high glucose and sodium pyruvate (DMEM, Thermo Fisher Scientific #31966047), supplemented with 10 mM HEPES buffer (Thermo Fisher Scientific #15630056), 1× non-essential amino acids (Thermo Fisher Scientific #11140035), penicillin-streptomycin (Thermo Fisher Scientific #15070063) and 10% fetal calf serum (Eurobio #CVFSVF00-01). Cells were maintained at 37°C and 5% CO_2_and passaged every 2–4 days at 1/10–1/2 dilutions. Cells were maintained for no more than 6 passages.

### Reagents

Human IFNAR blocking antibody (PBL Assay Science #21385–1) was used at a concentration of 5 μg/mL, ActD (Sigma-Aldrich #A1410) at 2 μg/μL. For protein extraction, Pierce BCA Protein Assay Kit (Thermo Fisher Scientific # 23225) was used following manufacturer’s instructions.

### Viruses

Influenza A/PR/8/76 (PR8) was purchased as purified allantoic fluid from Charles River Laboratories (Spafas, CT, USA). Plasmids coding for CHIKV-GFP and CHIKV-mCherry were purchased from EVA and obtained from the Andres Merits laboratory, respectively. SINV-GFP was derived from the pTR339 infectious clone. Plasmids were linearized overnight with *NotI* (Thermo Fisher Scientific # FD0593), then purified on columns (Macherey-Nagel #740609.50). *In vitro* transcription was performed using Ambion SP6 mMessage mMachine (Thermo Fisher Scientific #AM1340), according to manufacturer's instructions, and RNA was subsequently purified by phenol–chloroform extraction. Ten million BHK cells were electroporated with 10 μg of IVT RNA (1.2 kV, 25 μF, and infinite resistance) and put in culture with complete medium. Virus was harvested 72 h later, and was further passaged once for 24 h on BHK cells, before purification by ultracentrifugation to avoid protein contamination.

### Viral titers

Titers of the passage 1 virus stocks were determined by plaque assay on Vero cells as follows. Vero cells were seeded in 24-well plates at a confluence of 200,000 cells/well. 16–24 h later, they were infected with serial tenfold dilutions of the virus in DMEM for 1 h at 37°C, with gentle shaking after 30 min. Cells were then directly overlaid with 42°C-DMEM supplemented with 2% fetal calf serum and 0.8% agarose. 48 h post-infection, cells were fixed using 200 μL of 4% paraformaldehyde for 1 h, and revealed by crystal violet staining for 15 min at room temperature. Plaques were manually counted, and virus stock titers ranged 10^7^–10^8^.

### Infections

Unless otherwise stated, 150,000 cells were seeded in each well of a 12-well plate. 8–16 h later, they were incubated with virus diluted in 150 μL of serum-free complete medium at 37°C for 1 h at the indicated MOI, with gentle shaking after 30 min. Cells were then washed with 1 mL of PBS before fresh complete medium was added. Of note, MOIs were computed with respect to the number of cells plated, so are overestimated for late infections. MOIs used for the first infection were chosen as the three greatest powers of 10 that induced <50% cell death at the time of second infection.

### UV treatment and column filtering

Virus was dosed with 0.3 J cm^−2^ UV for 1 min using UVP CL-1000 Crosslinker (Thermo Fisher Scientific #UVP95022801) for inactivation. For filtering by column, Ultrafiltration Amicon Ultra 15 mL 100 kDa (Dutscher #044037) were used according to manufacturer's instructions, for 15–30 min at 4,000 × *g*.

### Flow cytometry

Cells were detached with trypsin–EDTA (Thermo Fisher Scientific, #25300054), centrifuged and washed once with complete medium and once with PBS. Cells were then stained with Violet Live/Dead marker (Thermo Fisher Scientific #10645203) at 1/500 dilution in PBS for 20 min at 4°C, then washed once with PBS and fixed with 100 μL of Cytofix/Cytoperm buffer (BD Biosciences #554714) for 20 min at room temperature, then resuspended in 200 μL of PBS. For IAV intracellular staining, cells were washed once with 1× Perm/Wash buffer (BD Biosciences #554714) and subsequently stained with rabbit FITC-conjugated anti-influenza A virus nucleoprotein antibody (Abcam #ab20921) at 1/100 in 1× Perm/Wash buffer for 45 min at 4°C. Cells were then washed twice with 1× Perm/Wash buffer and resuspended in 200 μL of PBS. Cells were imaged on a BD LSR II Fortessa, and analyzed with FlowJo v. X.0.7 (BD). Gating strategy is shown in S1 Fig.

### RNA extraction

Cells were trypsinized, washed with complete medium, then resuspended in 200 μL of PBS, then lysed and stocked at –80°C. RNA was subsequently extracted by High Pure RNA isolation (Roche, 11828665001) following manufacturer's instructions. For virus RNA extraction from supernatants, 200 μL of supernatant, cleared of cell debris by centrifugation at 2,000 rpm for 5 min at 4°C, was used instead of the 200 μL cell suspension in PBS.

### Reverse transcription and qPCR

Reverse transcription was performed using Maxima reverse transcriptase (Thermo Fisher Scientific #EP0741) and random primers (Thermo Fisher Scientific #SO142). qPCR was performed using Taqman Fast Advanced Master Mix (Thermo Fisher Scientific #4444557) according to the provider’s protocol. Taqman primer–probe mixes were used for quantification of mouse *Hprt* (Mm03024075_m1), *Ifnb1* (Mm00439552_s1) and *Cxcl10* (Mm00445235_m1). For detection of other genes, we designed the following primer–probe sets:

**Table:** 

**Gene**	**Forward primer**	**Reverse primer**	**Reporter probe**
GFP	CGTGCCCTGGCCCA	CACTGCACGCCGTAGGT	CCCTCGTGACCACCC
mCherry	GCTGAAGGTGACCAAGGGT	CTTGGAGCCGTACATGAACTGA	TCGCCTGGGACATCC
Genomic GFP	CATAACTTTGTACGGCGGTCCTA	CTCGCCCTTGCTCACCAT	CCGACAGCAAGTATC
Hamster *Hprt*	ACTGGAAAGAATGTCTTGATTGTTGAAGA	AGGAAAGCAAAGTCTGCATTGTT	TTGCCAGTGTCAATTAT

Custom gene expression assays were synthesized by Thermo Fisher Scientific. The StepOnePlus Real-Time PCR System (Thermo Fisher Scientific #4376600) was used for thermocycling and data acquisition. Thresholds were automatically determined by the StepOne Plus software, and corrected if they were outside the 0.1–1 range, as they otherwise allow for noise. Threshold count (C_T_) values were determined by the software and RNA levels were computed as 2^–(C_T_^gene^–C_T_^housekeeping^), and further normalized to the sample mean of the comparison group (white bar). RNA levels of samples for which C_T_ could not be determined were set to 0.

### RNA transfection

300,000 cells were seeded in each well of a 6-well plate, then infected as described above. Cells were then transfected with 5 μg of *in vitro* transcribed RNA (obtained as in the Viruses section) with Xfect RNA transfection reagent (Takarabio # 631450), following manufacturer’s instructions, in 1 mL of serum-free medium. Four hours post-transfection, medium was replaced with 2 mL of serum-containing growth medium, and control cells were washed three times with PBS and harvested to test for transfection efficiency.

### Generation of doxycycline-inducible constructs

Primers were designed to have a melting temperature around 60°C and synthesized from Integrated DNA Technologies. The vector used was pLVX-TRE3G-mCherry (Takara #631349) and the CHIKV coding sequence came from an IOL La Réunion strain infectious clone [45]. 50 μL PCR using Q5 2× Master Mix (New England Biolabs #M0492S) were performed using 20 or 100 ng of DNA template for the insert or the vector, respectively, and a final concentration of 0.25 μM of each primer, according to the following protocol: 30 s at 95°C, 18 cycles of 10 s at 95°C, 30 s at 65°C, 4.5 min at 72°C, and a final 5 min extension at 72°C. In each sample, 1 μL of FastDigest *DpnI* enzyme (Thermo Fisher Scientific #FD1703) was added, and they were incubated 2 h at 37°C prior to purification on columns (Macherey Nagel #740609). Inserts were cloned into the vector with In-Fusion HD cloning kit (Takara #638920) following manufacturer's instructions, and 2.5 μL of the reaction was transformed into 50 μL of Stellar Competent Cells (Takara #636766). The following day, individual colonies were grown in 2× YT media and mini-preps were subsequently performed (Macherey Nagel #740588). The presence of the insert was confirmed using Sanger sequencing.

### Transfection of the constructs

2.2 million 3T3 transactivator cells (Takara #631197) were plated in 10-cm plates. The following day, 8 μg of plasmid was transfected using JetPrime (Polyplus #114–07) following manufacturer's instructions. Twenty-four hours later, cells of each plate were trypsinized and divided into 6 wells at a confluence of 150,000 cells per well in 12-well plates in medium in the presence or absence of 1 μg/mL of doxycycline. Sixteen hours later, cells were challenged with CHIKV-GFP at an MOI of 4, as described above.

### Generation of nsP2 mutant

Using the IVA cloning approach [46], we simultaneously introduced two mutations (nsP2 S259P and R650D) into the CHIKV-GFP. Briefly, mutagenic primers were designed according to the IVA protocol, synthesized by Integrated DNA Technologies (IDT), and then used for mutagenic PCR with Q5 2× Master Mix (New England Biolabs #M0492S) instead of the Phusion polymerase. Following *DpnI* digestion (Thermo Fisher Scientific #FD1703) to remove residual wild-type plasmid, the PCR was transformed into Turbo competent cells (New England Biolabs #C2984I) and incubated at 30°C overnight. The following day, individual colonies were grown in 2× YT media and mini-preps were subsequently performed (Macherey Nagel #740588). The presence of mutations was confirmed using Sanger sequencing.

### Single-cell RNA-sequencing

HFF cells were plated in 6-well plates at a confluence of 300,000, and infected with CHIKV-GFP at an MOI of 1, then harvested as indicated above 24 h post-infection, with a long trypsin incubation (30 min at 37°C), to remove all virus bound to cells. After 2 washes with PBS supplemented with 0.04% BSA, 6,000 cells were processed on 10x genomics Single Cell 3’ v2 kit, following manufacturer’s instructions, using 12 cycles of cDNA amplification. Libraries were run on a NextSeq Mid output 150 cycles, and alignment and feature barcode matrix generation were performed using Cell Ranger (10x genomics). In total, ~168 million reads were sequenced, and 2,421 individual cells passed quality control, with a mean number of reads per cell of 69,576 and a median number of UMI counts per cell of 18,169. Subsequent analyses were performed using R v. 3.4.3 and cellrangerRkit package.

### Statistical analysis

Well treatments were not randomized on the plates and we were not blinded to any conditions. Correction for multiple testing was performed within each figure panel. Parametric tests were used throughout this study; although the normality assumption has not been tested—and cannot be tested with a quantified type II risk—, *t*-tests are known to be rather robust to non-normality [47]. Homoscedasticity was not clearly violated. Therefore, *t*-tests were used to compare two groups, and one-way analysis of variance followed by Dunnett's post-test to compare multiple groups. Comparisons were performed with respect to the control group indicated by the white bar.

## Supporting information

S1 Fig Gating strategy for flow cytometry analysis. Cells were isolated from debris, then single cells were gated, followed by live cells, out of which infected cells for each virus were assessed.
(TIFF)

S2 Fig Superinfection exclusion requires active replication, and is independent of the order of reporter virus addition. (**A**) BHK cells were infected with untreated or UV-irradiated CHIKV-GFP at the indicated MOI for 8 h, then with CHIKV-mCherry for another 8 h, before harvest and flow cytometry analysis. (**B**–**D**) BHK cells were infected with CHIKV-mCherry at the indicated MOI for 16 h then with CHIKV-GFP at MOI 1 for 8 h (B), and subsequently harvested and analyzed by flow cytometry (C, D). Bars indicate mean ± SD of biological triplicates, and data are representative of at least two independent experiments. NS, not significant; **p < 0.01, ***p < 0.001 (one-way analysis of variance followed by Dunnett’s post-test).
(TIFF)

S3 Fig CHIKV SIE is independent of nsP2 S259 and R650. (**A**) CHIKV-GFP(PD) was generated from CHIKV-GFP by mutation of two amino acids in the nsP2 protein. (**B**) BHK cells were infected with CHIKV-GFP or CHIKV-GFP(PD) at MOI 10^−3^ and GFP expression was monitored by flow cytometry for 48 h. (**C**, **D**) BHK cells were infected with CHIKV-GFP(PD) for 16 h at the indicated MOI then CHIKV-mCherry for 8 h at MOI 1, then analyzed by flow cytometry. Bars indicate mean ± SD of biological triplicates, and data are representative of at least two independent experiments. **p < 0.01, ***p < 0.001 (one-way analysis of variance followed by Dunnett’s post-test).
(TIFF)

S4 Fig qPCR primer–probe sets allow the specific monitoring of genomic and subgenomic challenge RNA replication. (**A**) The GFP primer–probe set targets the 162–212 region of the GFP gene, while the genomic GFP forward primer targets the last 27 bases of nsP4, the probe the 31–46 position of the subgenomic promoter, and the reverse primer the first 18 bases of GFP. (**B**) The mCherry primer–probe set targets the 161–237 region of the mCherry gene. (**C**,**D**) One million plaque forming unit (PFU) of CHIKV-GFP, CHIKV-mCherry or SINV-GFP were lysed. RNA was subsequently extracted and RT–qPCR was performed using the indicated primer–probe sets.
(TIFF)

S5 Fig SIE occurs at the replication level. (**A**, **B**) BHK cells were infected with CHIKV-mCherry for 16 h at the indicated MOI, then transfected with *in vitro* transcribed RNA coding for CHIKV-GFP. Twelve hours post-transfection, cells were harvested and analyzed by flow cytometry (A); 4 h post-transfection, transfection efficiency was controlled by RT–qPCR (B). (**C**) Fig 2E plotted in a logarithmic scale. (**D**) RNA upregulation between 1 and 8 h post-mCherry infection in samples infected by CHIKV-GFP at MOI 10^−2^. Bars indicate mean ± SD of biological triplicates, and data are representative of at least two independent experiments. NS, not significant; ***p < 0.001 (one-way analysis of variance followed by Dunnett’s post-test).
(TIFF)
