# Supplementary figures and images for: Chikungunya virus superinfection exclusion is mediated by a block in viral replication and does not rely on non-structural protein 2
Source: PLoS One. 2020 Nov 12;15(11):e0241592. doi: 10.1371/journal.pone.0241592 (PMC7660575; doi:10.1371/journal.pone.0241592)

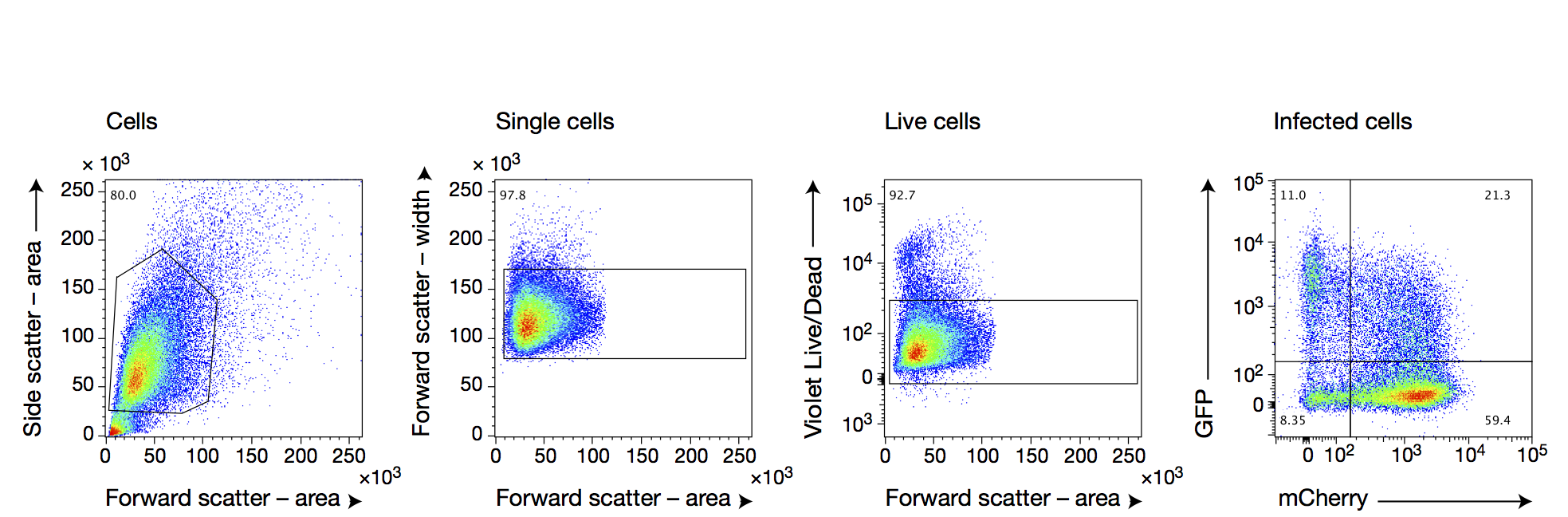

Supplement: S1 Fig — Cells were isolated from debris, then single cells were gated, followed by live cells, out of which infected cells for each virus were assessed. (TIFF) [file pone.0241592.s001.tiff]

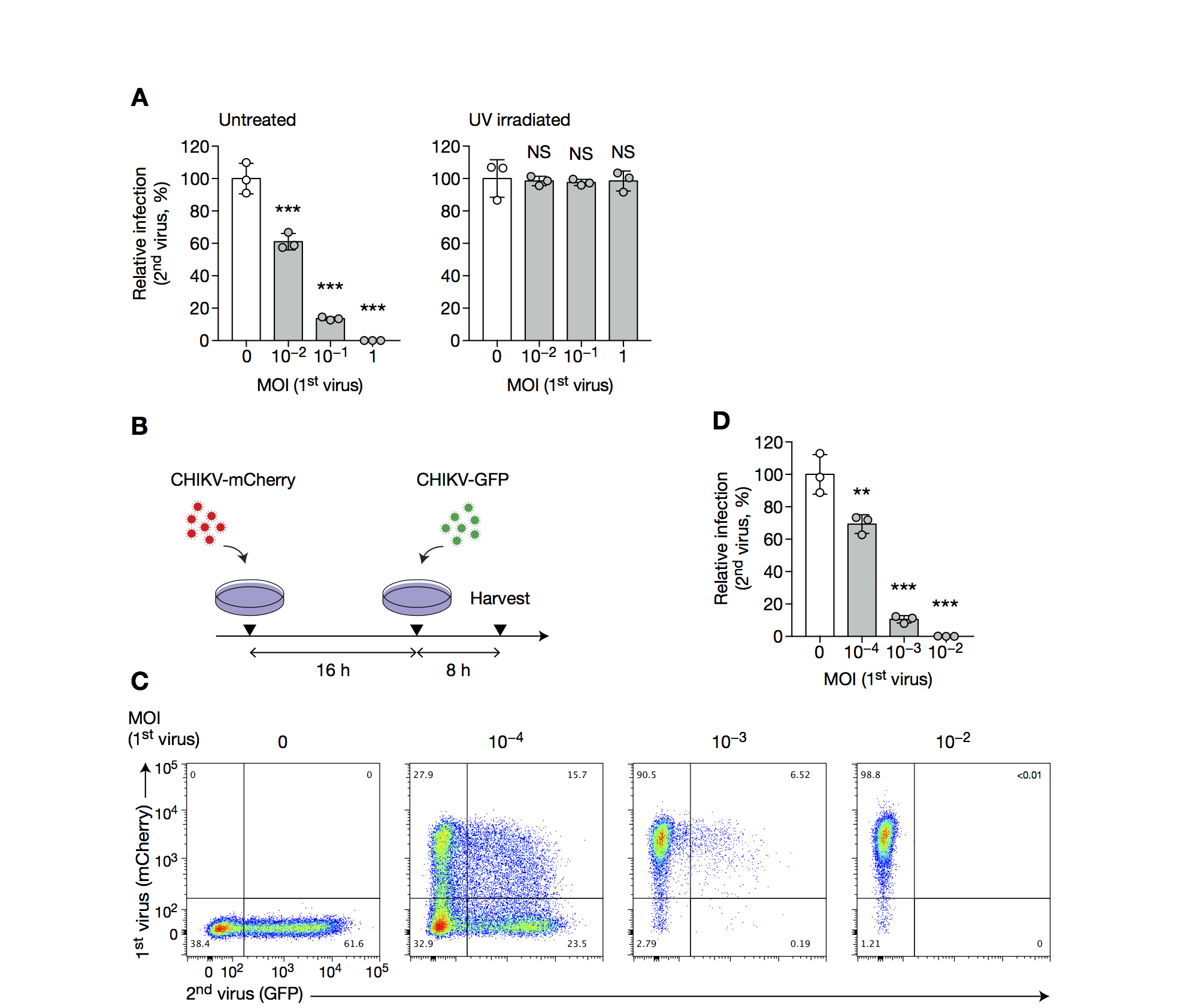

Supplement: S2 Fig — (A) BHK cells were infected with untreated or UV-irradiated CHIKV-GFP at the indicated MOI for 8 h, then with CHIKV-mCherry for another 8 h, before harvest and flow cytometry analysis. (B–D) BHK cells were infected with CHIKV-mCherry at the indicated MOI for 16 h then with CHIKV-GFP at MOI 1 for 8 h (B), and subsequently harvested and analyzed by flow cytometry (C, D). Bars indicate mean ± SD of biological triplicates, and data are representative of at least two independent experiments. NS, not significant; **p < 0.01, ***p < 0.001 (one-way analysis of variance followed by Dunnett’s post-test). (TIFF) [file pone.0241592.s002.tiff]

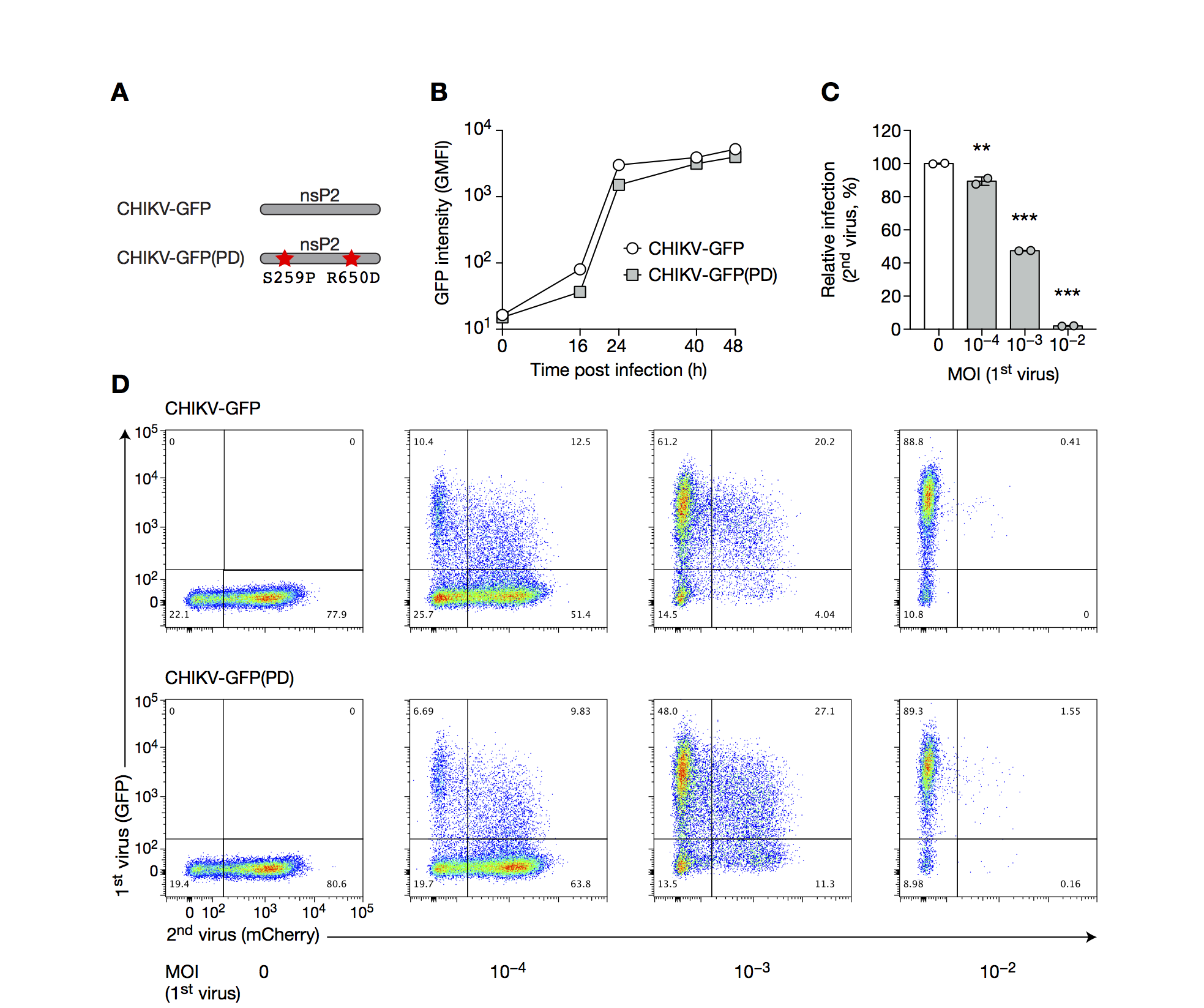

Supplement: S3 Fig — (A) CHIKV-GFP(PD) was generated from CHIKV-GFP by mutation of two amino acids in the nsP2 protein. (B) BHK cells were infected with CHIKV-GFP or CHIKV-GFP(PD) at MOI 10−3 and GFP expression was monitored by flow cytometry for 48 h. (C, D) BHK cells were infected with CHIKV-GFP(PD) for 16 h at the indicated MOI then CHIKV-mCherry for 8 h at MOI 1, then analyzed by flow cytometry. Bars indicate mean ± SD of biological triplicates, and data are representative of at least two independent experiments. **p < 0.01, ***p < 0.001 (one-way analysis of variance followed by Dunnett’s post-test). (TIFF) [file pone.0241592.s003.tiff]

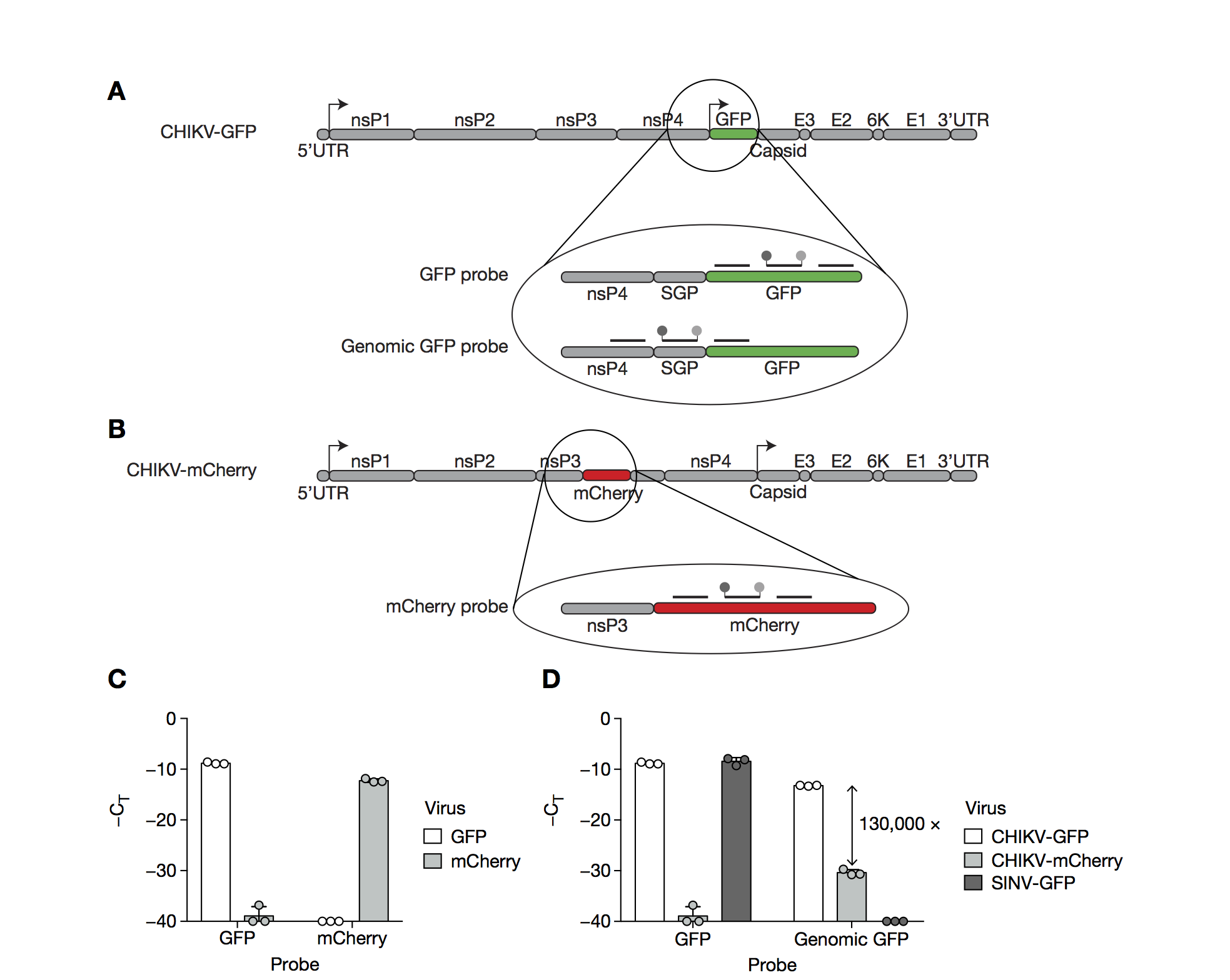

Supplement: S4 Fig — (A) The GFP primer–probe set targets the 162–212 region of the GFP gene, while the genomic GFP forward primer targets the last 27 bases of nsP4, the probe the 31–46 position of the subgenomic promoter, and the reverse primer the first 18 bases of GFP. (B) The mCherry primer–probe set targets the 161–237 region of the mCherry gene. (C,D) One million plaque forming unit (PFU) of CHIKV-GFP, CHIKV-mCherry or SINV-GFP were lysed. RNA was subsequently extracted and RT–qPCR was performed using the indicated primer–probe sets. (TIFF) [file pone.0241592.s004.tiff]

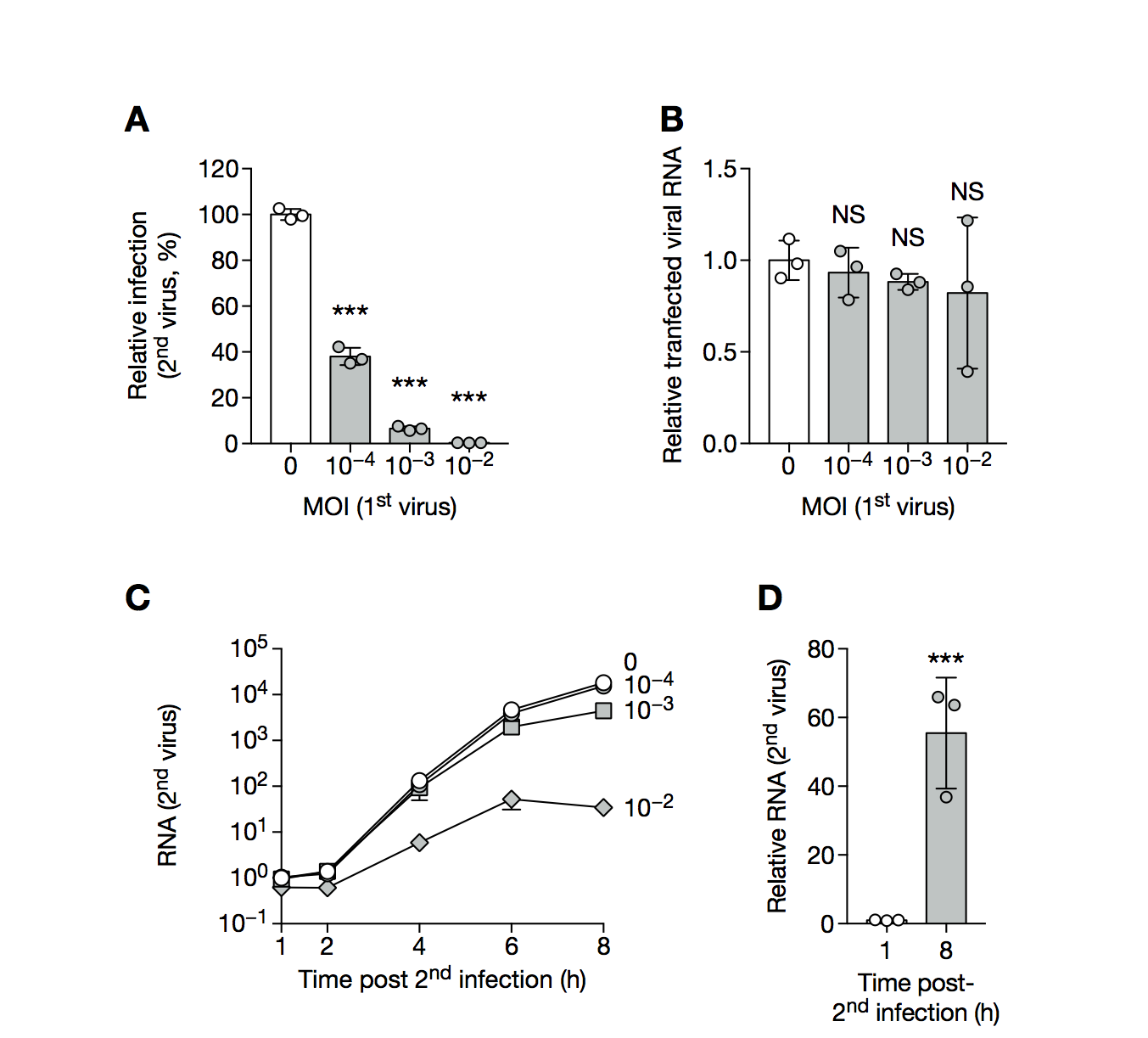

Supplement: S5 Fig — (A, B) BHK cells were infected with CHIKV-mCherry for 16 h at the indicated MOI, then transfected with in vitro transcribed RNA coding for CHIKV-GFP. Twelve hours post-transfection, cells were harvested and analyzed by flow cytometry (A); 4 h post-transfection, transfection efficiency was controlled by RT–qPCR (B). (C) Fig 2E plotted in a logarithmic scale. (D) RNA upregulation between 1 and 8 h post-mCherry infection in samples infected by CHIKV-GFP at MOI 10−2. Bars indicate mean ± SD of biological triplicates, and data are representative of at least two independent experiments. NS, not significant; ***p < 0.001 (one-way analysis of variance followed by Dunnett’s post-test). (TIFF) [file pone.0241592.s005.tiff]
